# Expression profile of androgen-modulated microRNAs in the fetal murine lung

**DOI:** 10.1186/s13293-016-0072-z

**Published:** 2016-04-01

**Authors:** Wafae Bouhaddioui, Pierre R. Provost, Yves Tremblay

**Affiliations:** Reproduction, Mother and Youth Health, Centre de Recherche du CHU de Québec, 2705 Laurier Boulevard, Rm T-3-67, Québec City, Québec Canada; Department of Obstetrics/Gynecology and Reproduction, Faculty of Medicine, Université Laval, Québec City, Québec Canada; Centre de Recherche en Biologie de la Reproduction (CRBR), Faculté de Médecine, Université Laval, Québec City, Québec Canada

**Keywords:** microRNA, Androgens, Preterm birth, Sex differences, Respiratory distress syndrome, Lung development, Surfactant

## Abstract

**Background:**

Androgens are known to delay lung development. As a consequence, the incidence and morbidity of respiratory distress syndrome of the neonate are higher for male than for female premature infants. We previously reported that many genes were expressed with a sex difference in the mouse developing lung and that several genes were under the control of androgens in the male fetal lung. microRNAs are small non-coding RNAs known to negatively regulate the expression of specific genes. In this study, we examined whether murine miRNAs are under the control of androgens in the male developing lung.

**Methods:**

Expression profiling of microRNAs was performed by microarrays using RNA extracted from male fetal lungs isolated on gestational day (GD) 17.0 and GD 18.0 after daily injection of pregnant mice from GD 10.0 with the antiandrogen flutamide or vehicle only. To identify putative miRNA target genes, the data obtained here were combined with gene profiling data reported previously using the same RNA preparations. qPCR was used to confirm microarray data with fetal lungs from other litters than those used in microarrays.

**Results:**

Flutamide induced downregulation and upregulation of several miRNAs on GD 17.0 and GD 18.0. Of the 43 mature miRNAs modulated by flutamide on GD 17.0, 60 % were downregulated, whereas this proportion was only of 34 % for the 35 mature miRNAs modulated on GD 18.0. For 29 and 26 flutamide-responsive miRNAs, we found a corresponding target inversely regulated by androgens on GD 17.0 and 18.0, respectively. The androgen-regulated target genes were involved in several biological processes (lipid metabolism, cell proliferation, and lung development) and molecular functions, mainly transcription factor binding.

**Conclusions:**

Regulation of male lung development involves several miRNAs that are under androgen modulation in vivo.

**Electronic supplementary material:**

The online version of this article (doi:10.1186/s13293-016-0072-z) contains supplementary material, which is available to authorized users.

## Background

Respiratory distress syndrome (RDS) is one of the most common complications of preterm babies [1]. The major cause of this disease is surfactant deficiency [2, 3], which is related to the immaturity of type II pneumonocytes (PTII), the surfactant-producing and -secreting cells [4]. Clinically, RDS is characterized by a sexual dimorphism with preterm boys more affected than preterm girls [5, 6]. This would be explained by a sexual difference in the timing of PTII cell maturation and the surge of surfactant synthesis leading to a disadvantage for male neonates [7–9]. Indeed, treatments in vitro and in vivo with the androgen dihydrotestosterone (DHT) or the antiandrogen flutamide demonstrated that androgens are responsible for the delay in PTII cell maturation and in the surge of surfactant synthesis in males [10, 11]. Additionally, experiments with testicular feminization mice (Tfm) showed that these negative actions of androgens occur via the androgen receptor (AR) [12] which is expressed in both male and female fetal lungs [13].

We previously reported that the expression profile of many genes presented a sexual dimorphism in the fetal lung at the end of the pseudoglandular [14], during the canalicular [14, 15], and at the beginning of the saccular stage of lung development [15]. Furthermore, we demonstrated that several genes were actively modulated by androgens in vivo on gestation days (GDs) 17.0 and 18.0 [15], a period including the surge of surfactant synthesis and the transition from the canalicular to the saccular stage. Since androgens have an important impact on the timing of lung maturation, it is important to deepen our knowledge on the regulatory mechanisms involved downstream of the activation of the androgen receptor.

miRNAs are small non-coding RNAs known to negatively regulate the expression of specific gene(s) by degrading mRNA(s) or inhibiting its/their translation into protein [16]. miRNAs are highly conserved across species [17]. They are involved in several physiological processes such as cell differentiation, proliferation, apoptosis, and lipid metabolism [18–21]. The importance of miRNAs in lung development was first demonstrated by Harris et al., who reported abnormal growth of epithelial tube, and an arrest of branching were observed in conditional knockout mice of *Dicer*, an important ribonuclease involved in biogenesis of mature miRNAs [22]. It was also demonstrated on GD 11.5 that two members of the Argonaute protein family, AGO1 and AGO2, were specifically expressed in lung distal epithelium and mesenchymal cells, respectively [23]. Therefore, these two RNA-induced silencing complex (RISC) components must be involved in cell-specific gene regulation. Finally, it was showed that miRNAs were dynamically regulated across lung development from the pseudoglandular to the alveolar stage [24].

Several miRNAs display a sexual dimorphism in their expression levels in different species [25–27]. Regulation of miRNA levels by sex steroids such as estradiol, progesterone, and testosterone has also been demonstrated [28–30]. A previous study reported sex differences in miRNA levels in the developing lung between GD 15.0 and GD 18.0 [31]. However, no study has examined sex steroid modulation of miRNA levels in fetal lungs. In the present study, using the antiandrogen flutamide, we investigated for the first time whether androgens modulate miRNAs expression in fetal murine lung during a developmental time overlapping the surge of surfactant synthesis.

## Methods

### Animals and housing

Protocols were approved by the Comité de Protection des Animaux du CHU de Québec (protocol no. 2011-053). Female and male Balb/c mice (Charles River Laboratories, Saint-Constant, QC, Canada) were housed with a 12-h light/dark cycle. Tap water and feed were provided ad libitum. Animals were mated in a 1-h mating window as previously described [15]. Pregnant females received a daily subcutaneous injection of 1 mg of the antiandrogen flutamide (kindly provided by Dr. Fernand Labrie) in 200 μl vehicle (0.9 % NaCl, 1 % gelatin (*w*/*v*) (ACP Chemicals, Saint-Léonard, QC, Canada), 10 % dimethylsulfoxide (Sigma-Aldrich, St. Louis, MO)) or vehicle only from GD 10.0 to the day prior to sacrifice. Pregnant females were sacrificed on GD 17.0 or GD 18.0 (term is GD 19.0) by exposure to CO_2_. GD 17.0 corresponded exactly to 17 days 0 h after the end of the 1-h mating window. From each fetus, the lungs and a rear leg were harvested, rapidly frozen on dry ice, and then stored at −80 °C until use.

### Fetal sex determination

Fetal sex was confirmed by PCR amplification of the male-specific *Sry* gene (GenBank: X67204) from fetal legs. DNAs were extracted with Extracta DNA Prep for PCR-Tissue (Quanta BioSciences). PCR reactions were performed using AccuStart PCR SuperMix Kit (Quanta BioSciences) according to the protocol of the manufacturer with 0.04 nM of each Sry primer (forward 5′-TATGGTGTGGTCCCGTGGTG-3′; reverse 5′-ATGTGATGGCATGTGGGTTCC-3′), resulting in a 282-nucleotide amplicon. The following PCR conditions were used: 94 °C for 5 min and 72 °C for 10 min followed by 34 cycles of 94 °C for 1 min, 65 °C for 1 min, and 72 °C for 1 min. Final extension was done at 72 °C for 10 min. Agarose gel electrophoresis was used for amplicon visualization.

### RNA extraction and sampling

Total RNA was extracted from the fetal lung of male subjects using TRI Reagent, a mixture of phenol and guanidine thiocyanate in a monophasic solution (Molecular Research Center, Cincinnati, OH, USA) and purified on a CsCl gradient as previously described [13]. The quality of RNA for microarray experiments was monitored by micro-capillary electrophoresis (Bioanalyzer 2100, Agilent Technologies, Santa Clara, CA, USA). For qPCR experiments, the RNA integrity was verified by gel electrophoresis. For all the experiments, RNA purity was determined using a Nanodrop 1000 spectrophotometer (Thermo Scientific). For all the samples, the OD 260/280 ratio was above 1.8. For microarray experiments, biological duplicates (*n* = 2 RNA pools) were prepared for each experimental condition with RNA from 4 to 5 male fetuses for each pool.

### Microarray experiments

Total RNA including low molecular weight RNA was labeled using the Flashtag RNA labeling kit (Affymetrix, Santa Clara, CA, USA) according to the manufacturer’s instructions. Briefly, for each sample, 400 ng of total RNA was subjected to a tailing reaction followed by the ligation of the biotinylated signal molecule to the target RNA sample. Each sample was hybridized to a GeneChip® miRNA Array 3.0 (Affymetrix, Santa Clara, CA, USA) for 16 h at 48 °C at 60 rpm. After washing and staining with a Fluidics Station 450 (Fluidics script FS450_0002), arrays were scanned with a GeneChip® Scanner 3000 7G (Affymetrix). The image data were analyzed with the Expression Console Software (www.affymetrix.com) for quality control. GeneChip® miRNA Array 3.0 contained 19,724 total mature miRNA probe sets and covered 153 organisms. This array contained 1111 and 855 mouse mature miRNA and pre-miRNA, respectively. miRNA probes of this array were derived from the Sanger miRBase miRNA database v17.

### Microarray data analysis

CEL files were imported and analyzed with the Partek Genomics Suite 6.6 software (Partek Incorporated, St. Louis, MO, USA). Background correction and normalization of probe set intensities were performed using the Robust Multiarray Analysis (RMA) method [32]. Quantile normalization was achieved and intensity values were Log_2_ transformed. Groups were compared by ANOVA analysis, and *p* values were corrected using the false discovery rate (FDR) procedure. The differences were considered statistically significant when *p* < 0.05 and FDR < 5 %. miRNAs were combined with their corresponding targets belonging to the GEO (GSE18135) data sets by Partek software based on the TargetScanMouse v6.2 algorithm. A chi-square test (2 × 2 table) was performed to analyze the significance of the variation in the proportions of miRNAs regulated by flutamide between GD 17.0 and GD 18.0.

### Reverse transcription and quantitative PCR

Reverse transcription and qPCR of miRNAs and normalization genes were performed as previously described [33]. Briefly, 200 ng of each RNA sample was denatured and mixed with 62.5 μM of each dNTP and 50 nM of the stem-loop primer at 65 °C for 5 min and then transferred on ice. First-strand buffer (SuperScript II kit, Life Technologies), 4 units of Protector RNase Inhibitor (Promega), and 50 units of SuperScript II RT (Life Technologies) were added to the mixture for a total reaction volume of 20 μl. Samples were incubated for 30 min at 16 °C, followed by pulsed reverse transcription of 60 cycles at 30 °C for 30 s, 42 °C for 30 s, and 50 °C for 1 s. Reverse transcriptase was then inactivated at 85 °C for 5 min. qPCR was performed using the FastStart Essential DNA Green qPCR Master Mix kits and a LC96 Instrument (Roche Diagnostics). Reactions were performed according to the manufacturer’s instructions with 0.5 μM of each primer and 20 ng of total RNA input in a final volume of 20 μl. Samples were incubated at 95 °C for 5 min, followed by 43 cycles of 95 °C for 5 s and 60 °C for 10 s. At the end of each run, samples were heated to 95 °C with a temperature transition rate of 0.2 °C/s to construct dissociation curves. The stability of five normalization genes was assessed by geNorm software as previously described [34]. The best combination of reference genes was used for relative quantification of microRNAs. The list of reverse transcription and qPCR primers for miRNAs and normalization genes are presented in Additional file 1: Table S1. The groups were compared using unpaired Student’s *t* test. The differences were considered statistically significant when *p* < 0.05.

## Results

### Androgen-regulated miRNA expression

**Fig. 1:**
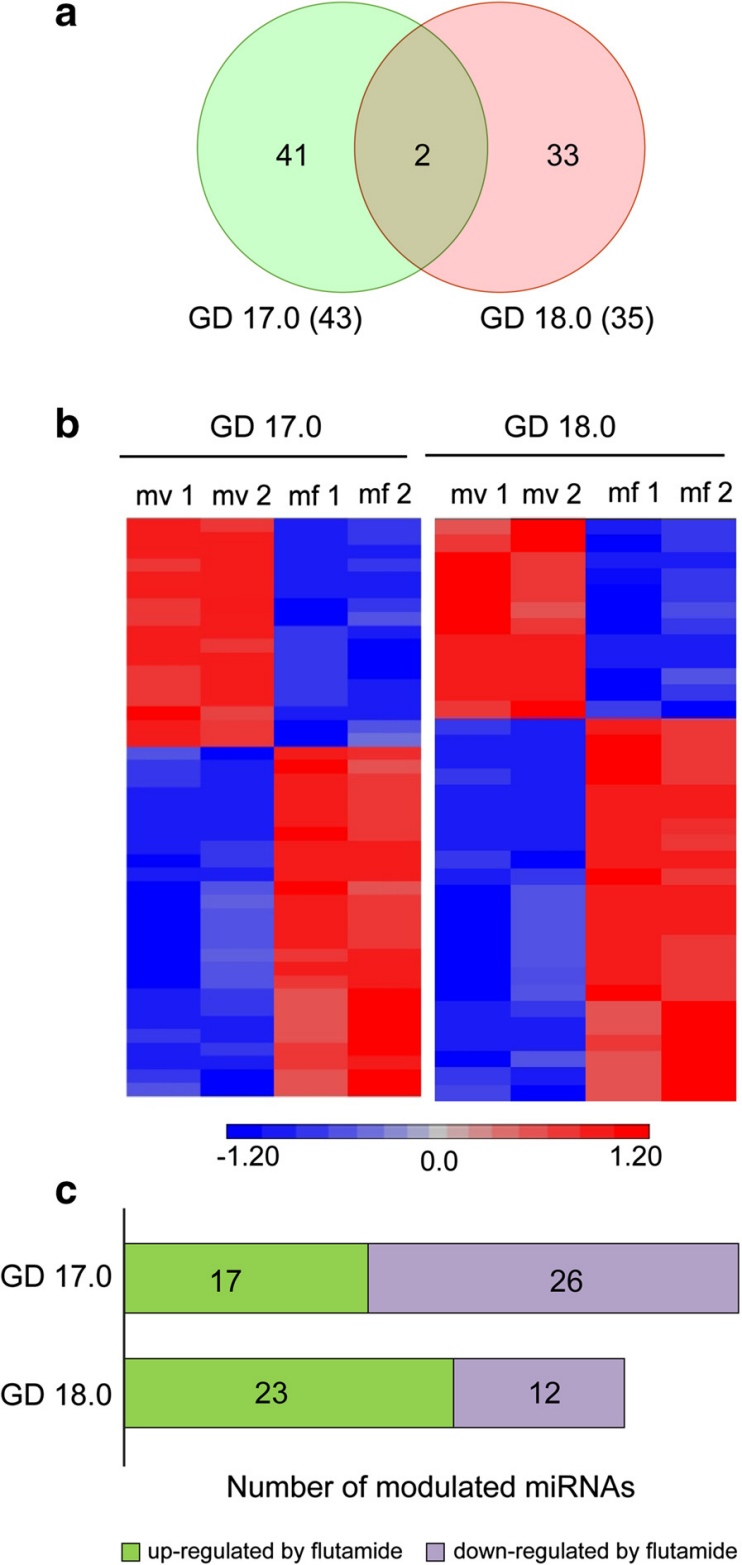
Overview of the microarray data obtained from fetal mouse lungs exposed or not to flutamide. **a** Number of androgen-regulated miRNAs on GD 17.0 and GD 18.0. **b** Expression profile of differentially expressed miRNAs is presented for each replica and each gestation time. *Red* and *blue* indicate the intensity level above and below the median, respectively, for each miRNA and each replicate. For each age, results are given for the two replicates (*1*, *2*) for male exposed to vehicle (*mv*), and male exposed to flutamide (*mf*). **c** Number of androgen-modulated mature miRNA according to their reactivity to flutamide at GD 17.0 and GD 18.0

Pregnant females were subjected to the antiandrogen flutamide or vehicle administration from GD 10.0 to the day prior sacrifice on GD 17.0 or 18.0. For each experimental condition, two biological replicates were studied. Each replicate contained lung RNA from several individual male fetuses belonging to different litters. Expression profiling of androgen-regulated miRNA was assessed by microarrays. For each gestation time, expression data from flutamide-treated males was compared with data from males injected with vehicle. Androgen-regulated miRNA were detected on GD 17.0 and GD 18.0 (Fig. 1a). Only two miRNAs overlapped the two gestational ages. The expression profile of differentially expressed miRNAs is presented for each replica (Fig. 1b). Of the 43 mature miRNAs modulated by flutamide on GD 17.0, 60 % were downregulated, whereas this proportion was only of 34 % for the 35 mature miRNAs modulated on GD 18.0 (Fig. 1c). This difference was statistically significant (chi-square 5.29, *p* = 0.021).

### qPCR analysis of microarray data

**Fig. 2:**
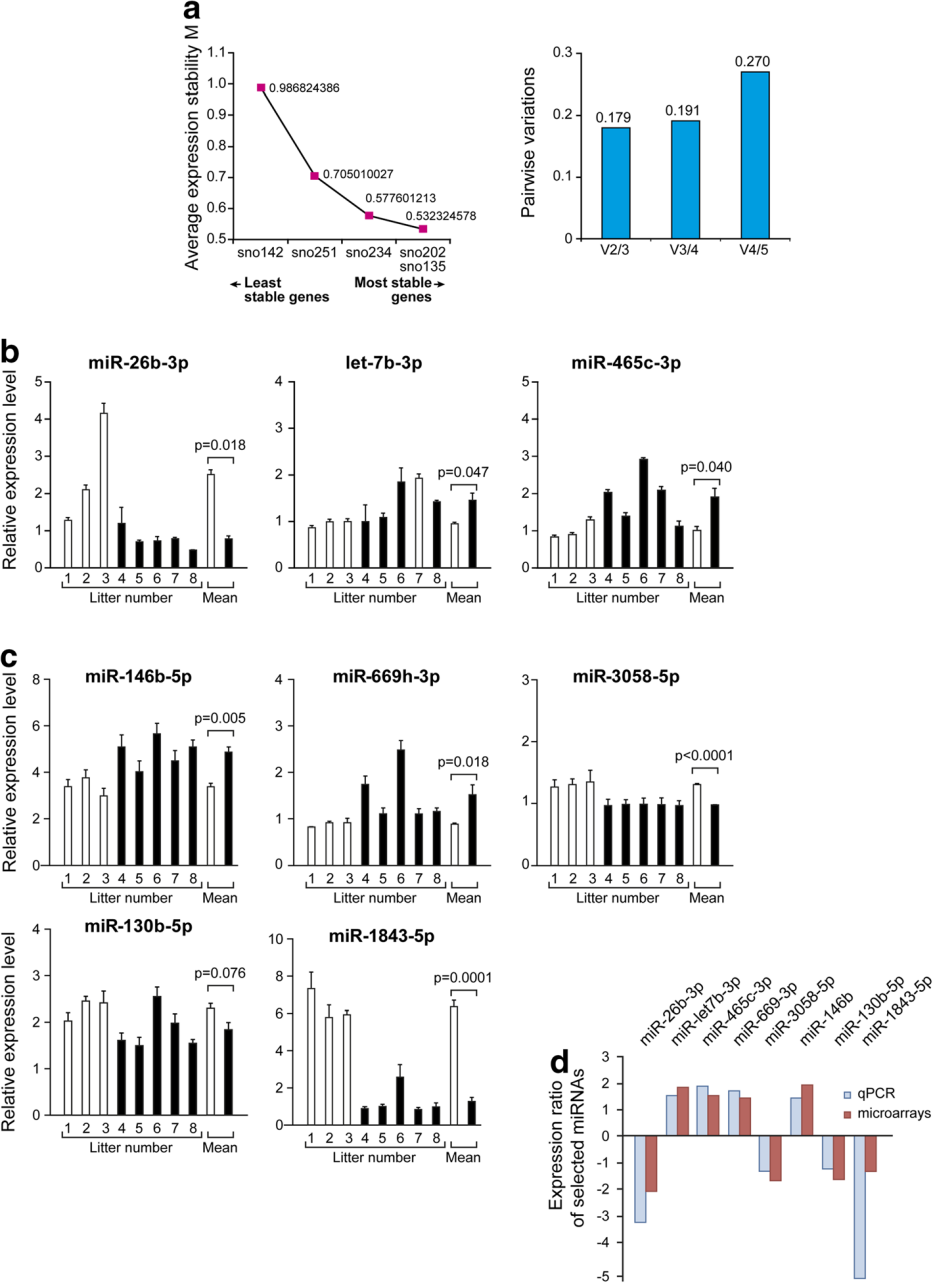
Validation of microarray data by qPCR for several miRNAs. **a** Selection of reference genes by geNorm among the five tested candidates. The average of expression stability M is presented for each tested reference gene candidate (*left panel*). The two most stable genes yielded the lower pairwise variation value (0.179) (*right panel*). Quantification of several miRNAs by qPCR on GD 17.0 (**b**) and GD 18.0 (**c**). **d** Comparison of expression ratios (flutamide/vehicle) obtained by microarray and qPCR for seven miRNA genes

After obtaining microarray data, animal breeding was reinitiated to produce independent RNA/cDNA samples to validate microarray data by qPCR. In contrast to RNA samples for microarrays, RNAs for qPCR were pooled by litter (one pool of male fetal lung RNA/litter). Three RNA pools were obtained for vehicle treatment for each age, while five pools were used for flutamide treatment for each age. Five reference genes were tested by geNorm: sno202, sno234, sno251, sno135, and sno142. Two were selected for normalization: sno202 and sno235 (Fig. 2a). Eleven miRNAs were randomly selected for qPCR analysis for each age: GD 17.0 miR-1843-5p, miR-485-3p, miR-711, miR-3962, miR-3067-3p, miR-212-3p, miR-669i, miR-877, miR-26b-3p, miR-465c-3p, let-7b-3p; GD 18.0 miR-1843-5p, miR-485-3p, miR-3473d, miR-132-5p, miR-3074-1-3p, miR-128-2-5p, miR-130b-5p, miR-490-5p, miR-669h-3p, miR-3058-5p, miR-146b. A statistically significant effect of flutamide was observed for seven of these miRNAs (miR-26b-3p, let-7b-3p, miR-465c-3p, miR-669h-3p, miR-3058-5p, miR-146b, miR-1843-5p), and a trend toward a statistically significant effect was observed for another miRNA (miR-130b-5p) (Fig. 2b, c). A statistically significant effect of flutamide was observed for miR-1843-5p on GD 18.0 but not on GD 17.0. For the other miRNAs, variations in expression levels from litter to litter prevented obtaining statistically significant differences (data not shown). The seven miRNAs presenting a statistically significant effect of flutamide and miR-130b-5p were used to compare qPCR and microarray data. All of them showed a similar effect of flutamide by qPCR and microarrays (Fig. 2d). According to the miRNA quality control (miRQC) study reported in 2014 [35], microarrays and qPCR approaches presented a concordance rate lower than 70 % for quantification of miRNA expression.

### Functional analysis of androgen-modulated miRNA

**Fig. 3:**
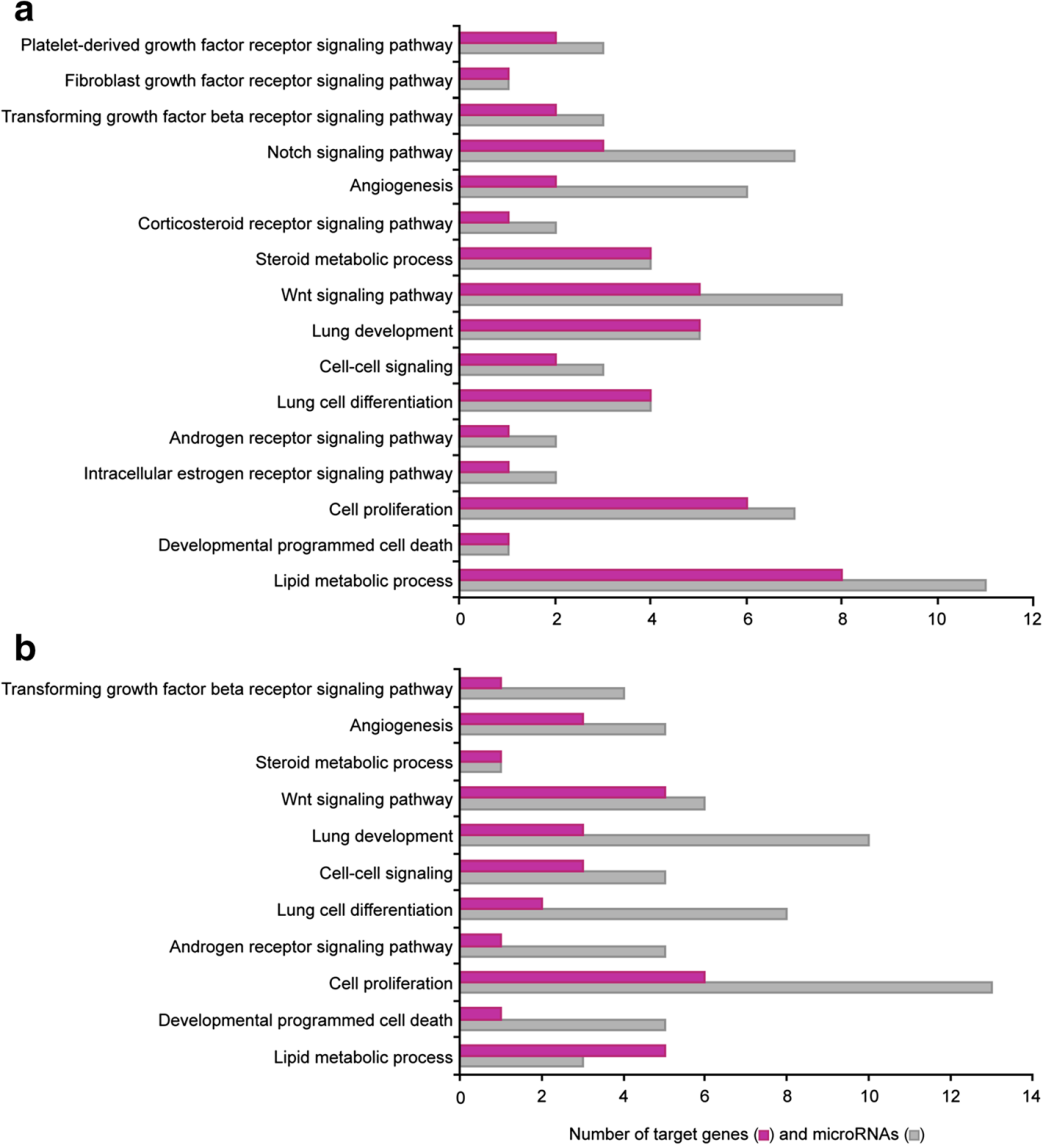
Number of androgen-regulated miRNAs and their potential androgen-regulated targets according to biological processes on GD 17.0 (**a**) and GD 18.0 (**b**)

**Fig. 4:**
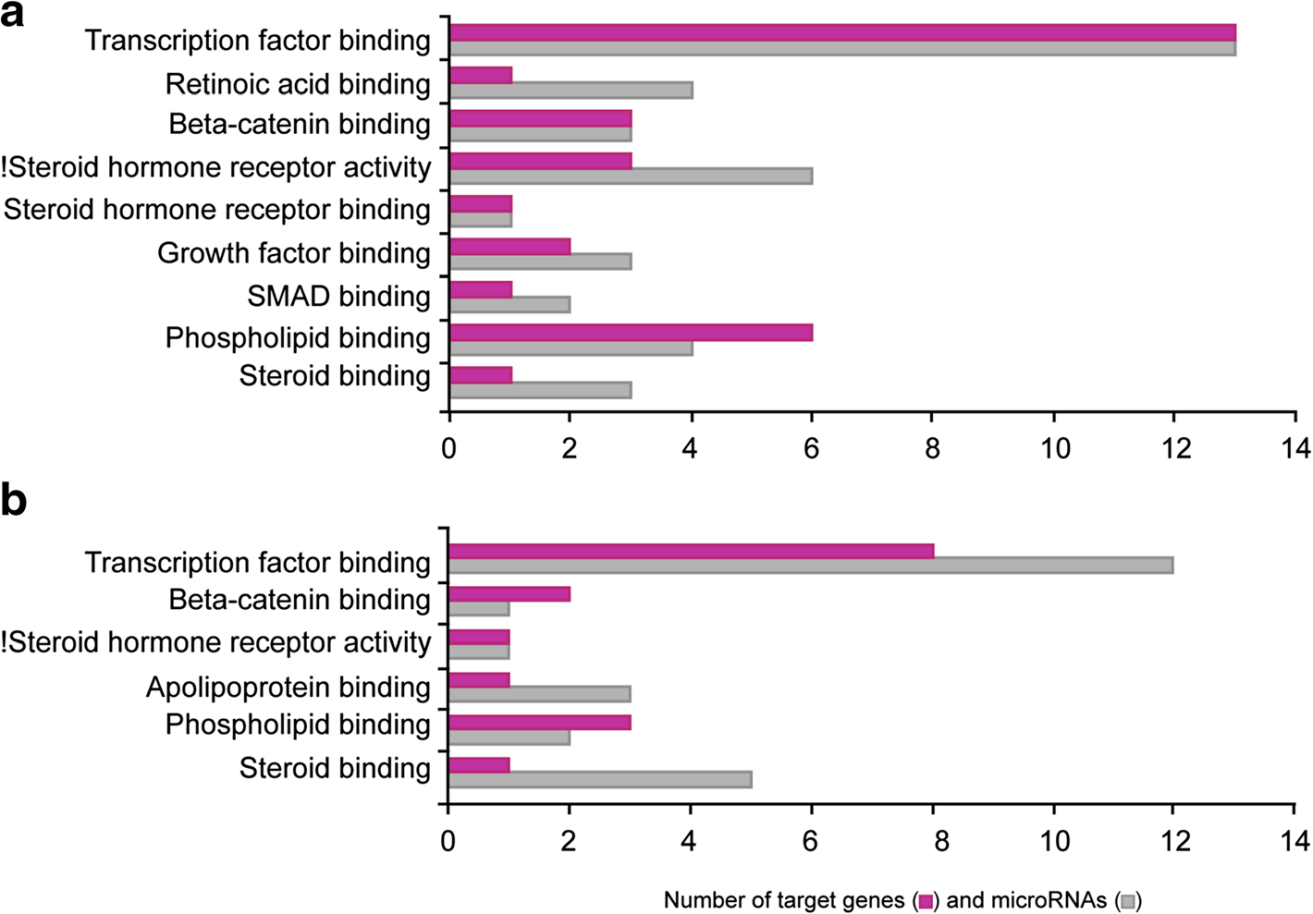
Number of androgen-regulated miRNAs and their potential androgen-regulated targets according to molecular functions on GD 17.0 (**a**) and GD 18.0 (**b**)

For microarrays, we utilized the RNA preparations that were used in our previous publication studying expression profiling of androgen-modulated genes [15]. Thus, the present miRNA profiling data were combined with the mRNA profiling data in order to pair miRNA with their potential targets showing an opposite regulation by flutamide. In silico miRNA-mRNA combination was performed. For 29 and 26 of the flutamide-responsive mature miRNAs, we found a corresponding target inversely regulated by androgens on GDs 17.0 and 18.0, respectively. Among all these mRNA targets, 25 were common to GD 17.0 and GD 18.0. Gene Ontology (GO) analysis was performed to determine biological processes (Fig. 3) and molecular functions (Fig. 4) involving androgen-regulated miRNAs and their corresponding targets. Among the biological processes, lipid metabolism, cell proliferation, lung development, Wnt signaling pathway, and angiogenesis showed the highest number of genes targeted by miRNAs for the two gestational ages (Fig. 3; Tables 1 and 2). For the molecular functions, transcription factor binding was the group with the highest number of targeted genes (Fig. 4) on GD 17.0 (Table 3) and GD 18.0 (Table 4).

**Table 1:** Androgen-regulated miRNAs and their corresponding mRNA target(s) on GD 17.0 classified according to several biological processes

Biological process	Gestational day 17.0	
	miRNA	mRNA target
Lipid metabolic process	*mmu-miR-3078*	**Etnk1; Pafah1b2**
	*mmu-miR-877*	**Elovl4; Etnk1**
	*mmu-miR-449a*	**Pdgfra**
	*mmu-miR-1955-5p*	**Hdlbp; Pik3r1**
	*mmu-miR-126-5p*	**Pbx1**
	*mmu-miR-362-3p*	**Pbx1**
	*mmu-miR-3470b*	**Smg1**
	*mmu-let-7f-2-3p*	**Col4a3bp; Etnk1**
	*mmu-let-7b-3p*	**Col4a3bp**
	*mmu-miR-1249-5p*	**Smg1**
	*mmu-miR-92a-1-5p*	**Hdlbp**
Developmental programmed cell death	*mmu-miR-449a*	**Slc4a7**
Cell proliferation	*mmu-miR-1306-5p*	**Zeb2**
	*mmu-miR-1955-5p*	**Gng2**
	*mmu-miR-215*	**Igf1; Zeb2**
	*mmu-miR-126-5p*	**Mmp16; Igf1; Yap1**
	*mmu-let-7f-2-3p*	**Col4a3bp**
	*mmu-let-7b-3p*	**Col4a3bp**
	*mmu-miR-674-3p*	**Mmp16**
Intracellular estrogen receptor signaling pathway	*mmu-miR-126-5p*	**Arid1a**
	*mmu-miR-3103-5p*	**Arid1a**
Androgen receptor signaling pathway	*mmu-miR-126-5p*	**Arid1a**
	*mmu-miR-3103-5p*	**Arid1a**
Lung cell differentiation	*mmu-miR-3078*	**Creb1**
	*mmu-miR-126-5p*	**Igf1**
	*mmu-miR-215*	**Igf2**
	*mmu-miR-687*	**Nfib**
Cell-cell signaling	*mmu-miR-3078*	**Gabra1; Gja1**
	*mmu-miR-1306-5p*	**Gja1**
	*mmu-let-7f-2-3p*	**Gabra1**
Lung development	*mmu-miR-126-5p*	**Igf1**
	*mmu-miR-215*	**Igf1**
	*mmu-miR-687*	**Nfib**
	*mmu-miR-449a*	**Pdgfra**
	*mmu-miR-3078*	**Zfpm2; Creb1**
Wnt signaling pathway	*mmu-miR-687*	**Apc**
	*mmu-miR-3089-3p*	**Tnks2**
	*mmu-miR-1843-5p*	**Sfrp1**
	*mmu-miR-449a*	**Tbl1xr1**
	*mmu-miR-3078*	**Tle4**
	*mmu-miR-1195*	**Tnks2**
	*mmu-miR-1249-5p*	**Sfrp1**
	*mmu-let-7b-3p*	**Tle4**
Steroid metabolic process	*mmu-miR-1955-5p*	**Hdlbp**
	*mmu-miR-126-5p*	**Pbx1**
	*mmu-miR-449a*	**Pdgfra**
	*mmu-miR-92a-1-5p*	**Hdlbp**
Corticosteroid receptor signaling pathway	*mmu-miR-126-5p*	**Arid1a**
	*mmu-miR-3103-5p*	**Arid1a**
Angiogenesis	*mmu-miR-362-3p*	**Naa15**
	**mmu-miR-467h**	*Map3k7*
	**mmu-miR-669i**	*Map3k7*
	*mmu-miR-1955-5p*	**Naa15**
	*mmu-miR-3078*	**Naa15**
	*mmu-miR-687*	**Naa15**
Notch signaling pathway	*mmu-miR-687*	**Adam10**
	*mmu-miR-3078*	**Adam17**
	*mmu-miR-3089-3p*	**Adam17**
	*mmu-miR-362-3p*	**Adam17**
	*mmu-miR-126-5p*	**Jag1**
	*mmu-miR-449a*	**Jag1**
	*mmu-let-7b-3p*	**Jag1**
Transforming growth factor beta receptor signaling pathway	*mmu-miR-3078*	**Creb1**
	**mmu-miR-467h**	*Map3k7*
	**mmu-miR-669i**	*Map3k7*
Fibroblast growth factor receptor signaling pathway	**mmu-miR-149**	*Ndst1*
Platelet-derived growth factor receptor signaling pathway	*mmu-miR-449a*	**Pdgfra**
	*mmu-miR-3089-3p*	**Zfand5**
	*mmu-miR-126-5p*	**Zfand5**

Italicized data are upregulated by flutamide; boldface data are downregulated by flutamide

**Table 2:** Androgen-regulated miRNAs and their corresponding mRNA target(s) on GD 18.0 classified according to several biological processes

Biological process	Gestational day 18.0	
	miRNA	mRNA target
Lipid metabolic process	*mmu-miR-30e*	**B3gnt5; St8sia4; Nr5a2; Myo5a**
	*mmu-miR-146b*	**Myo5a**
	*mmu-miR-361-3p*	**Smg1**
Developmental programmed cell death	*mmu-miR-30e*	**Bcl2**
	*mmu-miR-202-5p*	**Bcl2**
	*mmu-miR-3058*	**Bcl2**
	*mmu-miR-669h-3p*	**Bcl2**
	*mmu-miR-703*	**Bcl2**
Cell proliferation	*mmu-miR-130a*	**Appl1; Zeb2**
	*mmu-miR-146b*	**Appl1**
	*mmu-miR-452-3p*	**Appl1**
	*mmu-miR-202-5p*	**Bcl2**
	*mmu-miR-3058*	**Bcl2**
	*mmu-miR-703*	**Bcl2**
	*mmu-miR-3473d*	**Bcl2l2**
	*mmu-miR-669h-3p*	**Hdgfrp3; Bcl2**
	*mmu-miR-1251*	**Igf1**
	*mmu-miR-344f-3p*	**Igf1**
	*mmu-miR-709*	**Myc; Igf1**
	*mmu-miR-466l-5p*	**Igf1**
	*mmu-miR-30e*	**Igf1; Bcl2; Zeb2**
Androgen receptor signaling pathway	*mmu-miR-146b*	**Med13**
	*mmu-miR-3473d*	**Med13**
	*mmu-miR-669h-3p*	**Med13**
	*mmu-miR-703*	**Med13**
	*mmu-miR-883a-3p*	**Med13**
Lung cell differentiation	*mmu-miR-1251*	**Igf1**
	*mmu-miR-30e*	**Igf1**
	*mmu-miR-344f-3p*	**Igf1; Creb1**
	*mmu-miR-466l-5p*	**Creb1; Igf1**
	*mmu-miR-709*	**Igf1**
	*mmu-miR-130a*	**Creb1**
	*mmu-miR-202-5p*	**Creb1**
	*mmu-miR-703*	**Creb1**
Cell-cell signaling	*mmu-miR-883a-3p*	**Gls**
	*mmu-miR-130a*	**Lrp6**
	*mmu-miR-709*	**Lrp6**
	*mmu-miR-30e*	**Myo5a; Lrp6**
	*mmu-miR-146b*	**Myo5a**
Lung development	*mmu-miR-344f-3p*	**Igf1; Creb1**
	*mmu-miR-466l-5p*	**Igf1**
	*mmu-miR-202-5p*	**Creb1**
	*mmu-miR-703*	**Creb1**
	*mmu-miR-130a*	**Creb1**
	*mmu-miR-1251*	**Foxp2; Igf1**
	**mmu-miR-3058**	*Igf1; Foxp2*
	*mmu-miR-490-5p*	**Foxp2; Igf1**
	*mmu-miR-709*	**Foxp2; Igf1**
	*mmu-miR-669h-3p*	**Foxp2; Igf1**
	*mmu-miR-30e*	**Igf1**
Wnt signaling pathway	*mmu-miR-669h-3p*	**Apc**
	*mmu-miR-130a*	**Lrp6**
	*mmu-miR-30e*	**Lrp6**
	*mmu-miR-709*	**Myc; Lrp6**
	*mmu-miR-105*	**Usp34**
	**mmu-miR-669b-3p**	*Mitf*
Steroid metabolic process	*mmu-miR-30e*	**Nr5a2**
Angiogenesis	*mmu-miR-30e*	**Pdcd10**
	*mmu-miR-202-5p*	**Naa15**
	*mmu-miR-3085-5p*	**Naa15**
	*mmu-miR-883a-3p*	**Naa15**
	*mmu-miR-669h-3p*	**Srpk2**
Transforming growth factor beta receptor signaling pathway	*mmu-miR-130a*	**Creb1**
	*mmu-miR-202-5p*	**Creb1**
	*mmu-miR-344f-3p*	**Creb1**
	*mmu-miR-703*	**Creb1**

Italicized data are upregulated by flutamide; boldface data are downregulated by flutamide

**Table 3:** Androgen-regulated miRNAs and their corresponding mRNA target(s) on GD 17.0 classified according to molecular functions

Molecular function	Gestational day 17.0	
	miRNA	mRNA target
Steroid binding	*mmu-miR-126-5p*	**Igf1**
	*mmu-miR-215*	**Igf1**
Phospholipid binding	*mmu-miR-126-5p*	**Ogt; Pitpnb**
	**mmu-miR-291a-3p**	*Wdr45*
	*mmu-let-7f-2-3p*	**Col4a3bp**
	*mmu-let-7b-3p*	**Pik3c2a; Sbf2; Col4a3bp**
SMAD binding	*mmu-miR-1306-5p*	**Zeb2**
	*mmu-miR-215*	**Zeb2**
Growth factor binding	*mmu-miR-215*	**Col5a1**
	*mmu-miR-449a*	**Pdgfra**
	*mmu-miR-1249-5p*	**Col5a1**
Steroid hormone receptor binding	*mmu-miR-1955-5p*	**Pik3r1**
Steroid hormone receptor activity	**mmu-miR-291a-3p**	*Nr2f2*
	*mmu-miR-362-3p*	**Nr2c2**
	**mmu-miR-467b**	*Nr2f2*
	**mmu-miR-467d**	*Nr2f2*
	**mmu-miR-467h**	*Nr2f2*
	*mmu-miR-432*	**Rorb**
Beta-catenin binding	*mmu-miR-687*	**Apc**
	*mmu-miR-449a*	**Tbl1xr1**
	*mmu-let-7f-2-3p*	**Cd2ap**
Retinoic acid binding	**mmu-miR-291a-3p**	*Nr2f2*
	**mmu-miR-467b**	*Nr2f2*
	**mmu-miR-467d**	*Nr2f2*
	**mmu-miR-467h**	*Nr2f2*
Transcription factor binding	*mmu-miR-3078*	**Creb1; Tle4; Meis2; Ddx3x**
	*mmu-miR-465c-3p*	**Ddx3x**
	**mmu-miR-291a-3p**	*E2f2*
	**mmu-miR-467b**	*E2f2*
	**mmu-miR-467d**	*E2f2*
	**mmu-miR-467h**	*E2f2*
	**mmu-miR-212-3p**	*Foxo3*
	*mmu-miR-362-3p*	**Kdm5c; Trip12**
	*mmu-miR-432*	**Kdm5c; Rorb**
	*mmu-miR-1195*	**Meis2**
	*mmu-miR-126-5p*	**Nfya; Zeb1; Ppargc1a; Pbx1**
	*mmu-miR-687*	**Nfya**
	*mmu-let-7b-3p*	**Tle4**

Italicized data are upregulated by flutamide; boldface data are downregulated by flutamide

**Table 4:** Androgen-regulated miRNAs and their corresponding mRNA target(s) on GD 18.0 classified according to several molecular functions

Molecular function	Gestational day 18.0	
	miRNA	mRNA target
Steroid binding	*mmu-miR-1251*	**Igf1**
	*mmu-miR-30e*	**Igf1**
	*mmu-miR-344f-3p*	**Igf1**
	*mmu-miR-466l-5p*	**Igf1**
	*mmu-miR-709*	**Igf1**
Phospholipid binding	*mmu-miR-130a*	**Ccdc88a**
	*mmu-miR-30e*	**Eea1; Nr5a2**
Apolipoprotein binding	*mmu-miR-130a*	**Lrp6**
	*mmu-miR-30e*	**Lrp6**
	*mmu-miR-709*	**Lrp6**
Steroid hormone receptor activity	*mmu-miR-30e*	**Nr5a2**
Beta-catenin binding	*mmu-miR-669h-3p*	**Apc**
	*mmu-miR-669h-3p*	**Cd2ap**
SMAD binding	*mmu-miR-30e*	**Zeb2**
	*mmu-miR-130a*	**Zeb2**
	*mmu-miR-666-5p*	**Zeb2**
Transcription factor binding	*mmu-miR-202-5p*	**Bcl2; Creb1**
	*mmu-miR-30e*	**Bcl2**
	*mmu-miR-703*	**Bcl2; Med13; Creb1**
	*mmu-miR-130a*	**Bptf; Tcf4; Rbbp8; Creb1**
	*mmu-miR-3473d*	**Bptf; Med13**
	*mmu-miR-466l-5p*	**Bptf; Tcf4**
	*mmu-miR-709*	**Tcf4; Myc**
	*mmu-miR-344f-3p*	**Creb1**
	*mmu-miR-669h-3p*	**Bcl2; Nfyb; Med13**
	*mmu-miR-883a-3p*	**Med13**
	*mmu-miR-146b*	**Med13**
	*mmu-miR-452-3p*	**Tcf4**

Italicized data are upregulated by flutamide; boldface data are downregulated by flutamide

The following miRNAs, shown to be involved in lung development, were not modulated by flutamide in our experiment: miR-221 [36], miR-429/200a/200b/200c/141 from the miR-200 family [37], miR-150 [38], miR-142 [39], miR-127 [40], miR-375 [41], and miR-26a [42].

## Discussion

The importance of miRNAs in lung development has been reported in several studies [43]. Knowing that androgens modulate lung development [9, 44], we investigated the possibility that several androgen effects be mediated by the regulation of miRNAs. We demonstrate for the first time in this report that the levels of several miRNAs are modulated by androgens in late developing lungs on GDs 17.0 and 18.0. These two gestational days overlap the transition period from the canalicular to the saccular stages. The canalicular stage is characterized by the formation of distal airway bronchioles, epithelial differentiation into type I and type II pneumonocytes, and the beginning of angiogenesis. The saccular stage includes the formation of terminal saccules, epithelial cell thinning, and growth of capillary networks. Between GDs 17.0 and 18.0, important changes in lung morphogenesis occur, which are accompanied by variations in the expression of several genes. These dynamic changes are compatible with our data showing a different profile of androgen-modulated miRNAs for each age, with only two miRNAs in common between the two gestational days. In fact, 76 miRNAs were modulated by androgens. Of these, 41 and 33 were specifically modulated on GD 17.0 and GD 18.0, respectively.

We previously demonstrated that 1597 and 1775 genes were modulated by flutamide on GD 17.0 and GD 18.0, respectively, in the fetal mouse lung [15]. Of these, only 590 and 428 genes, respectively, presented a sex difference [15]. Based on the demonstration that steroidogenic enzymes involved in the synthesis and inactivation of androgens are expressed in the developing lung of both sexes, a role for androgens was proposed in lung development of females as well as males [13, 45, 46]. This does not exclude sex differences originating most probably from circulating androgens of testicular origin. Levels of androgen-regulated miRNAs are dependent on androgen receptor activation, which is affected by the availability of androgens, which in turn is affected by circulating amounts of androgens and by local expression of steroidogenic enzymes. One androgen-synthesizing enzyme, 17β-hydroxysteroid dehydrogenase (HSD) type 5 (GenBank accession no. AH007907), and one androgen-inactivating enzyme, 17β-HSD type 2 (NM_008290), are both expressed in the mouse developing lung at levels that were shown to vary according to developmental time and from litter to litter [13]. As a consequence, the amplitude of the effect of androgens on miRNA expression is likely to vary from litter to litter. Therefore, sampling may induce variations in the levels of androgen-regulated miRNAs. qPCR experiments were designed not only to study the effect of flutamide but also to evaluate the reproducibility from litter to litter. Our data indicate that the effect of flutamide observed from the population cannot always be observed for individual litters.

The design of our study did not allow identification of target genes of miRNAs acting through the inhibition of translation but rather of those targeting mRNA stability. The particular interest of our miRNA profiling experiment is that we used the same RNA samples as those used for gene profiling of the effect of flutamide by Bresson et al. [15]. Therefore, we combined the data from the two profiling studies to select miRNA-regulated target genes showing an opposite modulation by flutamide compared to their corresponding miRNAs. Flutamide inhibits specifically the androgen receptor response. Positive and negative effects of androgens are inhibited by flutamide, leading to an apparent downregulation or upregulation of gene expression, respectively. Since each gene may be subjected to several miRNAs and other regulatory factors, the putative target genes of a given miRNA are not all expected to be inversely regulated compared to this miRNA. Nevertheless, our approach identified combinations of miRNAs and their mRNA targets that were inversely modulated by flutamide. The combination of the two profiling studies revealed that the target genes inversely regulated by flutamide compared to miRNAs were involved in several biological processes and molecular functions relevant to lung development such as lipid metabolism, cell proliferation and differentiation, cell-cell signaling, several signaling pathways, angiogenesis, and more as presented in Figs. 3 and 4.

Two miRNAs among the 20 most abundant miRNAs detected in the fetal lung on GD 17.5 [47] were downregulated by androgens (upregulated by flutamide) on GD 17.0 in our study, namely, miR-92a-1-5p and miR-449a. miR-92a-1-5p belongs to the miR-17/92 cluster involved in lung morphogenesis and overexpressed in the early stage of lung development [47]. Accordingly, mice deficient for miR17/92 die shortly after birth with lung hypoplasia and cardiac defects [48]. Transducin (beta)-like 1X-related protein 1 (TBL1XR1 or TBLR1) mRNA is a predictive target for miR-449a that is conversely regulated by androgens. The corresponding protein is involved in β-catenin binding since it is essential for the recruitment of β-catenin for Wnt-β-catenin-mediated transcription [49]. Then, downregulation of miR-449a by androgens and, consequently, upregulation of TBL1XR1 should positively regulate Wnt-β-catenin gene expression on GD 17.0.

miR-449a is also known to target jagged 1 (jag1) mRNA, which encodes the ligand of the Notch 1 receptor. It was previously demonstrated that the miR-449 family contributes to cell fate determination by targeting the Notch signaling pathway [50]. Our results showed that jag1 mRNA is also regulated by two other miRNAs (miR-126-5p and let-7b-3p) that, like miR-449a, were upregulated by flutamide. Moreover, two other genes also involved in the Notch signaling pathway, ADAM10 and ADAM17, were upregulated by androgens like jag1, and their miRNAs were upregulated by flutamide (Table 1). These data strongly suggests that the Notch signaling pathway should be under androgen regulation on GD 17.0. Notch proteins and their ligands are highly expressed in lung development [51, 52] since they are required for the differentiation of epithelial cells, more specifically ciliated cells, in the bronchial epithelium. Our data strongly suggest that these effects of Notch signaling may be regulated positively by androgens.

It was reported that the Let-7 family, including let-7f-2-3p and let-7b-3p, are the most abundant miRNAs in the fetal lung on GD 17.5 [47]. It was shown that fetal lung expression levels of mmu-let-7b-3p were lower in early than in late developmental stages [40]. Two members of the Let-7 family, let-7f-2-3p and let-7b-3p, were downregulated by androgens in the present study. In addition to jag1 mRNA mentioned above, the messenger of transducin-like enhancer of split 4 (*Tle4*) is also a let-7b-3p putative target gene downregulated by flutamide. *Tle4* is known to be expressed in embryonic stem cells where it acts as a repressor of cell pluripotency and self-renewal, thus favoring cell differentiation [53]. Given that the Tle4 mRNA was downregulated by flutamide in our experiments, androgens may exert a positive pressure in the favor of cell differentiation through let-7b-3p.

Our data showed that several androgen-modulated genes involved in growth factor signaling [15] are targeted by androgen-modulated miRNAs. Among these, insulin-like growth factor 1 gene (*IGF1*) was under positive regulation by androgens through miR-215 on GD 17.0, and through miR-30e, miR-1251, miR-709, miR-344f-3p, and miR-466l-5p on GD 18.0. IGF1 is involved in cell proliferation and distal epithelium differentiation in prenatal lung [54]. Interestingly, *IGF1* expression was upregulated in RDS [55], which presented a higher incidence and morbidity for males, whereas we show in this report that IGF1 mRNA levels were upregulated by androgens. Moreover, IGF2 mRNA levels were also positively modulated by androgens. It is a putative target of miR-215, which in turn is negatively regulated by androgens. As demonstrated by the study of *IGF2*^−/−^ mice, a lack of IGF2 leads to a delay in lung maturation characterized by a dense pseudoglandular-like appearance on GD 17.5 [56].

Nuclear receptor 2 factor 2 (*Nr2f2*), also known as chicken ovalbumin upstream promoter-transcription factor II (*COUP-TFII*), antagonizes retinoic acid (RA) signaling in the developing lung, allowing the formation of distal lung structures under the regulation of FGF10 and BMP4 [57]. Our data showed that COUP-TFII mRNA levels were downregulated by androgens on GD 17.0 but not on GD 18.0 [15]. In this report, some miRNAs putatively targeting the COUP-TFII mRNA were conversely regulated by androgens on GD 17.0: miR-291a-3p, miR-467b and miR-467d, and miR467h. Therefore, these miRNAs must participate in the negative regulatory pressure of androgens on the expression of *COUP-TFII* and thus cause a delay in the formation of distal lung structures.

miR-130a was shown to be expressed in murine fetal lung, where a decrease in miR-130a levels was observed from GD 15.0 to GD 17.0, followed by an increase up to GD 18.0 [36]. It was observed that pulmonary localization of this miRNA changed according to gestational age and corresponded on GD 18.0 to terminal bronchioles and mesenchymal cells around developing saccules [36]. Upregulation of miR-130a was shown to increase vascular density and distal airway branching [36]. In contrast, downregulation of miR-130a with an anti-miR led to reduced airway branching in the lung explant [36]. Our data indicated that miR-130a levels were upregulated by flutamide on GD 18.0. One putative miR-130a target is cAMP response element binding protein 1 (Creb1) mRNA. *Creb1* is also regulated by flutamide and plays an important role in the differentiation of epithelial cells, mainly type I epithelial cells [58]. Our data suggest that androgens exert both a positive pressure on the expression of *Creb1* and a negative pressure on vascularization and distal airway branching on GD 18.0 through miR-130a. Another putative target of miR-130a is Zeb2 mRNA. This messenger was downregulated by flutamide on GD 18.0 in our previous gene profiling study [15]. Zeb2 repressed transcription of E-cadherin and, consequently, epithelial cell polarity and adhesion [59]. Zeb2 was also shown to be involved in type II epithelial cell differentiation [37]. Therefore, upregulation of Zeb2 by androgens may impact these mechanisms.

Some miRNAs modulated by flutamide in our study have also been shown to be regulated by hyperoxia, which is a model of bronchopulmonary dysplasia [60, 61]. Let-7f-2-3p (GD 17.0), miR-30e (GD 18.0), and miR-709 (GD 18.0) were upregulated by flutamide in our experiment, whereas they were upregulated by hyperoxia during the postnatal period [60, 61]. In contrast, miR-146b was upregulated by flutamide but downregulated by hyperoxia [61]. Knowing that a sex difference was reported in the incidence of bronchopulmonary dysplasia (BPD) [62–64] and that this sex difference may originate from androgens, it would be interesting to test whether the miRNAs regulated by androgens at ages cited above are involved in the sex difference observed in BPD.

## Conclusions

The levels of several miRNAs are modulated by androgens in the developing lung on GDs 17.0 and 18.0 thus during the transition from the canalicular to the saccular stage. Comparison with data from our previous study on the effect of the antiandrogen flutamide on the genomics of the developing lung allowed pairing of several androgen-regulated miRNAs with their androgen-regulated putative target(s). Putative miRNA target genes belonged to several biological processes and functions important for lung development.

## Additional file

Additional file 1: Table S1. List of primers used for reverse transcription and qPCR for selected miRNA genes. (DOCX 20 kb)
