# Supplementary material for: Expression profile of androgen-modulated microRNAs in the fetal murine lung
Source: Biol Sex Differ. 2016 Apr 1;7:20. doi: 10.1186/s13293-016-0072-z (PMC4818395; doi:10.1186/s13293-016-0072-z)
Supplement: Additional file 1: Table S1. — List of primers used for reverse transcription and qPCR for selected miRNA genes. (DOCX 20 kb) [file 13293_2016_72_MOESM1_ESM.docx]

**Table S1 List of primers used for reverse transcription and qPCR for selected miRNA genes**

| **Gene** | **Accession number** | **Reverse transcription primer/**  **qPCR forward primer^c^**  **(5’-3’)** |
| --- | --- | --- |
| mmu-miR-26b-3p | MIMAT0004630^a^ | GTTGGCTCTGGTGCAGGGTCCGAGGTATTCGCACCAGAGCCAACGAGCCA |
|  |  | GCGGCGGCCTGTTCTCCATTACT |
| mmu-let-7b-3p | MIMAT0004621^a^ | GTTGGCTCTGGTGCAGGGTCCGAGGTATTCGCACCAGAGCCAACGGGAAG |
|  |  | GCGGCGGCTATACAACCTACTGC |
| mmu-miR-465c-3p | MIMAT0004874^a^ | GTTGGCTCTGGTGCAGGGTCCGAGGTATTCGCACCAGAGCCAACTCTACT |
|  |  | GCGGCGGGATCAGGGCCTTTCTA |
| mmu-miR-146b-5p | MIMAT0003475^a^ | GTTGGCTCTGGTGCAGGGTCCGAGGTATTCGCACCAGAGCCAACAGCCTA |
|  |  | GCGGCGGTGAGAACTGAATTCCA |
| mmu-miR-669h-3p | MIMAT0005842^a^ | GTTGGCTCTGGTGCAGGGTCCGAGGTATTCGCACCAGAGCCAACTGTGCA |
|  |  | GCGGCGGTATGCATATACACACA |
| mmu-miR-3058-5p | MIMAT0014813^a^ | GTTGGCTCTGGTGCAGGGTCCGAGGTATTCGCACCAGAGCCAACTCTTCC |
|  |  | GCGGCGGTCAGCCACGGCTTACCT |
| mmu-miR-130b-5p | MIMAT0004583^a^ | GTTGGCTCTGGTGCAGGGTCCGAGGTATTCGCACCAGAGCCAACAGTAGT |
|  |  | GCGGCGGACTCTTTCCCTGTTGC |
| mmu-miR-485-3p | MIMAT0003129^a^ | GTTGGCTCTGGTGCAGGGTCCGAGGTATTCGCACCAGAGCCAACGAGAGG |
|  |  | GCGCGCGAGTCATACACGGCTCT |
| mmu-miR-711 | MIMAT0003501^a^ | GTTGGCTCTGGTGCAGGGTCCGAGGTATTCGCACCAGAGCCAACCTTACA |
|  |  | GCGGCGGGGGACCCGGGGAGAGA |
| mmu-miR-3962 | MIMAT0019340^a^ | GTTGGCTCTGGTGCAGGGTCCGAGGTATTCGCACCAGAGCCAACAAATGT |
|  |  | GCGGCGGAGGTAGTAGTTTGT |
| mmu-miR-3067-3p | MIMAT0014841^a^ | GTTGGCTCTGGTGCAGGGTCCGAGGTATTCGCACCAGAGCCAACCCTCTC |
|  |  | GCGGCGGCCAAGCGGCTGCCCTGG |
| mmu-miR-212-3p | MIMAT0000659^a^ | GTTGGCTCTGGTGCAGGGTCCGAGGTATTCGCACCAGAGCCAACTGGCCG |
|  |  | GCGGCGGTAACAGTCTCCAGTCA |
| mmu-miR-669i | MIMAT0005840^a^ | GTTGGCTCTGGTGCAGGGTCCGAGGTATTCGCACCAGAGCCAACGTATGC |
|  |  | GCGGCGGTGCATATACACACAT |
| mmu-miR-877 | MIMAT0004861^a^ | GTTGGCTCTGGTGCAGGGTCCGAGGTATTCGCACCAGAGCCAACCCCTGC |
|  |  | GCGGCGGGTAGAGGAGATGGC |
| mmu-miR-3473d | MIMAT0020632^a^ | GTTGGCTCTGGTGCAGGGTCCGAGGTATTCGCACCAGAGCCAACAAGGGC |
|  |  | GCGGCGGCCACTGAGCCACTTTCCA |
| mmu-miR-132-5p | MIMAT0016984^a^ | GTTGGCTCTGGTGCAGGGTCCGAGGTATTCGCACCAGAGCCAACGTAACA |
|  |  | GCGGCGGAACCGTGGCTTTCGAT |
| mmu-miR-3074-1-3p | MIMAT0014857^a^ | GTTGGCTCTGGTGCAGGGTCCGAGGTATTCGCACCAGAGCCAACCGGTGC |
|  |  | GCGGCGGGATATCAGCTCAGTAG |
| mmu-miR-128-2-5p | MIMAT0017069^a^ | GTTGGCTCTGGTGCAGGGTCCGAGGTATTCGCACCAGAGCCAACTCTCTT |
|  |  | GCGGCGGGGGGGCCGATGCACTGT |
| mmu-miR-130b-5p | MIMAT0004583^a^ | GTTGGCTCTGGTGCAGGGTCCGAGGTATTCGCACCAGAGCCAACAGTAGT |
|  |  | GCGGCGGACTCTTTCCCTGTTGC |
| mmu-miR-490-5p | MIMAT0017261^a^ | GTTGGCTCTGGTGCAGGGTCCGAGGTATTCGCACCAGAGCCAACACCCAC |
|  |  | GCGGCGGCCATGGATCTCCAG |
| mmu-miR-1843-5p | MIMAT0014805 | GTTGGCTCTGGTGCAGGGTCCGAGGTATTCGCACCAGAGCCAACAGTCAG |
|  |  | GCGGCGGTATGGAGGTCTCTGTC |
| sno-202 | AF357327^b^ | GTTGGCTCTGGTGCAGGGTCCGAGGTATTCGCACCAGAGCCAACCATCAG |
|  |  | GCGGCGGGCTGTACTGACTTGA |
| sno-142 | AF357324^b^ | GTTGGCTCTGGTGCAGGGTCCGAGGTATTCGCACCAGAGCCAACTTCCTC |
|  |  | GCGGCGGGTCAGTGCCACGTGT |
| sno-135 | AF357323^b^ | GTTGGCTCTGGTGCAGGGTCCGAGGTATTCGCACCAGAGCCAACCTTCAG |
|  |  | GCGGCGGCTAAAATAGCTGGAA |
| sno-251 | AF357332^b^ | GTTGGCTCTGGTGCAGGGTCCGAGGTATTCGCACCAGAGCCAACCTGGCT |
|  |  | GCGGCGGATACATACTTGCCCT |
| sno-234 | AF357329^b^ | GTTGGCTCTGGTGCAGGGTCCGAGGTATTCGCACCAGAGCCAACTCTCAG |
|  |  | GCGGCGGCTTTTGGAACTGAAT |

^a^ : miRBase accession number

^b^ : NCBI accession number

^c^ :The universal reverse primer for qPCR is the same for all the genes: 5’-GTGCAGGGTCCGAGGT-3’
